# Precise measurement for line structure light vision sensor with large range

**DOI:** 10.1038/s41598-023-34428-w

**Published:** 2023-05-04

**Authors:** Wei-wei Sheng

**Affiliations:** School of Science, Qingdao University of Technology, Qingdao, 266520 China

**Keywords:** Optical techniques, Optics and photonics, Applied optics, Optical sensors

## Abstract

High precision and large measurement range are the target of any one three-dimensional scanner. For a line structure light vision sensor, measurement precision depends on its calibration results, i.e., determining mathematical expression of the light plane in camera coordinate system. However, as calibration results are locally optimal solutions, high precise measurement in a large range is difficult. In this paper, we give a precise measurement method and the corresponding calibration procedure for a line structure light vision sensor with a large measurement range. A motorized linear translation stages with a travel range of 150 mm and a planar target which is a surface plate with a machining precision of 0.05 mm are utilized. With the help of the linear translation stage and the planar target, functions which gives the relationship between center point of the laser stripe and the perpendicular/ horizontal distance are obtained. Once image of light stripe is captured, we can get a precise measurement result from the normalized feature points. Compared with a traditional measurement method, distortion compensation is not necessary and precision of measurement is improved significantly. Experiments show that root mean square error of measurement results according to our proposed method is reduced by 64.67% related to the traditional method.

## Introduction

A three-dimensional line structure light vision sensor (*LSLVS*) is normally consisted of one image sensor and a line laser projector. It is widely used in the area of industrial measurement owing to its wide measurement range, high precision, easy information extraction and so on. These *LSLVS*s can be classified into two categories according to their construction.

In the first category, the image sensor is a normal camera with a normal lens^1,2^, i.e. the image plane is parallel to the lens plane. The relationship between image sensor and laser projector is unchangeable and triangulate in the process of measurement. Spatial points can be confirmed once the relationship is determined, which is known as calibration of *LSLVS*.

Heretofore, there are many calibration methods for *LSLVS*. These methods can be classified into three categories according to the ways of obtaining feature points on the laser plane: 3D target based method, planar target based method and 1D target based method^3^.

In the 3D target based method, geometrical features have been widely used in recent years. Xiao et al.^4^ used an additional facility to control the 3D target, i.e. a very precisely metal cube, to move in pure translation accurately in the purpose of obtaining a vanishing point of the structured light plane, and then the projection angle of the light plane projector was solved from the vanishing point, as well as the baseline, the intercept of structured light plane on *x*-axis of the image coordinate system. Yang et al.^5^ got two parallel lines on the structured light plane by using a 3D target with two precisely visible parallel planes, when several vanishing points were obtained, the normal vector of the structured light plane could be deduced. As the baseline was solved based on the invariance of cross-ratio, calibration of the structured light plane was accomplished. Unfortunately, 3D target based method^6–8^ is not accurate enough because of the problem of mutual occlusion between different planes of the target and fewer feature points. Additionally, the 3D target, normally a cube with some special accessories, is difficult to make precisely and cumbersome for on-site calibration.

The planar target based method is more available to calibrate LSLVS. Wei et al.^9,10^ utilized a planar target with checkerboard pattern to finish the calibration. Based on the invariance of double cross-ratio, intersection points of the light stripe and checkerboards can be obtained under the image coordinate system as the exactly known size of each checkerboard. Then enough feature points on the light plane can be obtained. According to related fitting algorithm, expression of the light plane under the camera coordinate system can be calculated out. Liu et al.^11^ proposed a new method according to Plücker matrix to represent the light stripe on a planar target. When the target is located in several different positions, Plücker matrixes of light stripes can be obtained. Then expression of the light plane can be solved by combining obtained Plücker matrixes. Wei et al.^12^ calibrate a LSLVS based on vanishing feature. Vanishing points of the light plane could be obtained from intersection point of the light stripe and vanishing line of the target plane. Once the planar target is moved to enough different positions, the normal vector of the light plane could be calculated out as well as the vanishing line. As the size of the planar target is exactly known, the parameter *D* could be deduced consequently. Then function of the light plane under camera coordinate system was determined.

Compared with 3D target based method and planar target based method, 1D target based method^13^ is proposed owing to its convenient operation. Normally, feature points on the light plane can be obtained based on related algorithm, such as the intersection point of the light stripe and a 1D target, which can be obtained based on the invariance of cross-ratio. By moving the 1D target randomly to more different positions, enough feature points can be obtained to fit the light plane.

As the relative relation between the sensor and the laser projector is not request strictly as the sensor in the first category, this kind of sensor is cheap and convenient. Unfortunately, captured images over the whole measurement range are not sharp enough to get a precise measurement result, especially in the z-direction (height direction). In other words, measurement range of this kind of *LSLVS* in height direction is limited.

In the second category, relationship between the image sensor and laser projector satisfies the *Scheimpflug* condition strictly^14^, i.e., the CCD plane, the lens plane and the focus plane (normally a laser plane) intersect in a single line, which is named as *Scheimpflug* line. In this case, measurement range is enlarged. As requirement of the precise machining, this kind of *LSLVS* is expensive which is often utilized as a commercial sensor such as *KEYENCE LJ-X*8000, *COGNEX DS*910*B* and so on. Moreover, calibration method is difficult for this kind of *LSLVS*. Shao et al^15^ give a mathematic model to define the camera with a tilted lens. Then the measurement mode for the *LSLVS* in *Scheimpflug* conditions is given. When the target with circle pattern is located in the measurement range, the *LSLVS* can be calibrated. But as the location/pose of the target is limited, precision of the calibration is not high enough.

So how to enlarge the measurement range and execute an easy calibration method to get precise measurement results are significant, which are also the purpose of all kinds of 3D laser scanners. In this work, we propose a measurement method for the *LSLVS* in traditional construction, including its calibration method. Compared with a traditional measurement method, distortion compensation is not necessary and precision of measurement is improved significantly. Moreover, this approach can also be used to the *LSLVS* in *Scheimpflug* conditions.

## Model of a LSLVS

### LSLVS in traditional structure

**Figure 1:**
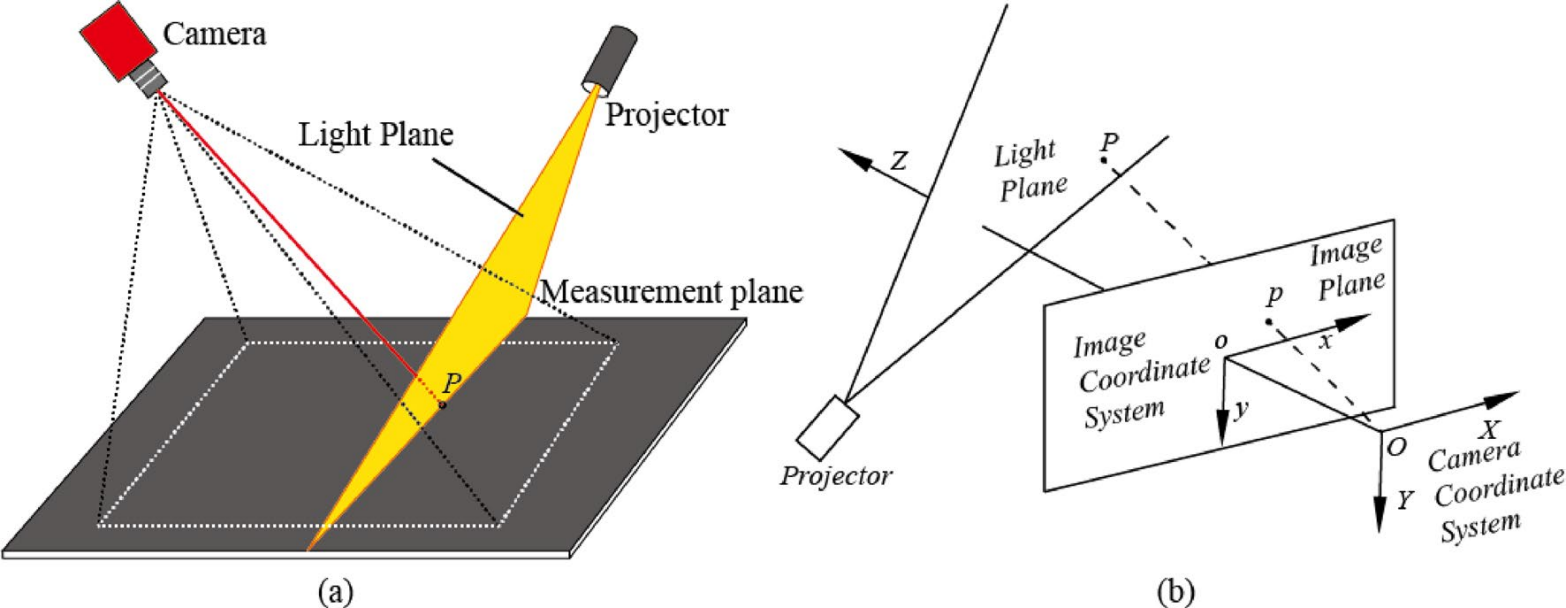
(**a**) The typical structure of a LSLVS and (**b**) its measurement model.

A typical structure of a *LSLVS* in traditional structure is illustrated in Fig. 1a, while its corresponding measurement model is in Fig. 1b. As illustrated in Fig. 1a, a laser projector projects a laser stripe onto surface of the measuring object. Images of the laser stripe are captured by the camera. According to the measurement model (which is illustrated in Fig. 1b), 3D coordinates of feature points on the laser plane under the camera coordinate system can be calculated out.

In Fig. 1b, O*-XYZ* is the Camera Coordinate System (*CCS*) while *o-xy* is the Image Coordinate System (*ICS*). Under the *CCS*, center of the camera is at the origin and the optical axis points to the positive *Z* direction. A spatial point P is projected onto the plane with *Z* = *f*_0_, referred to as the image plane under the *CCS*, where *f*_0_ is the effective focal length (*EFL*). Supposing *p* = (*x*, *y*, 1)^T^ is the projection of *P* = (*X*, *Y*, *Z*)^T^ on the image plane. Under the idealized pinhole imaging model, *P*, *p* and center of the camera *O* are collinear. Relationship between the camera and the laser projector remains unchangeable in the process of measurement^12^.

Traditionally, determining the relationship (i.e. mathematical expression of the laser plane under the *CCS*) is very important which is known as calibration of a *LSLVS*.

### LSLVS in Scheimpflug conditions

**Figure 2:**
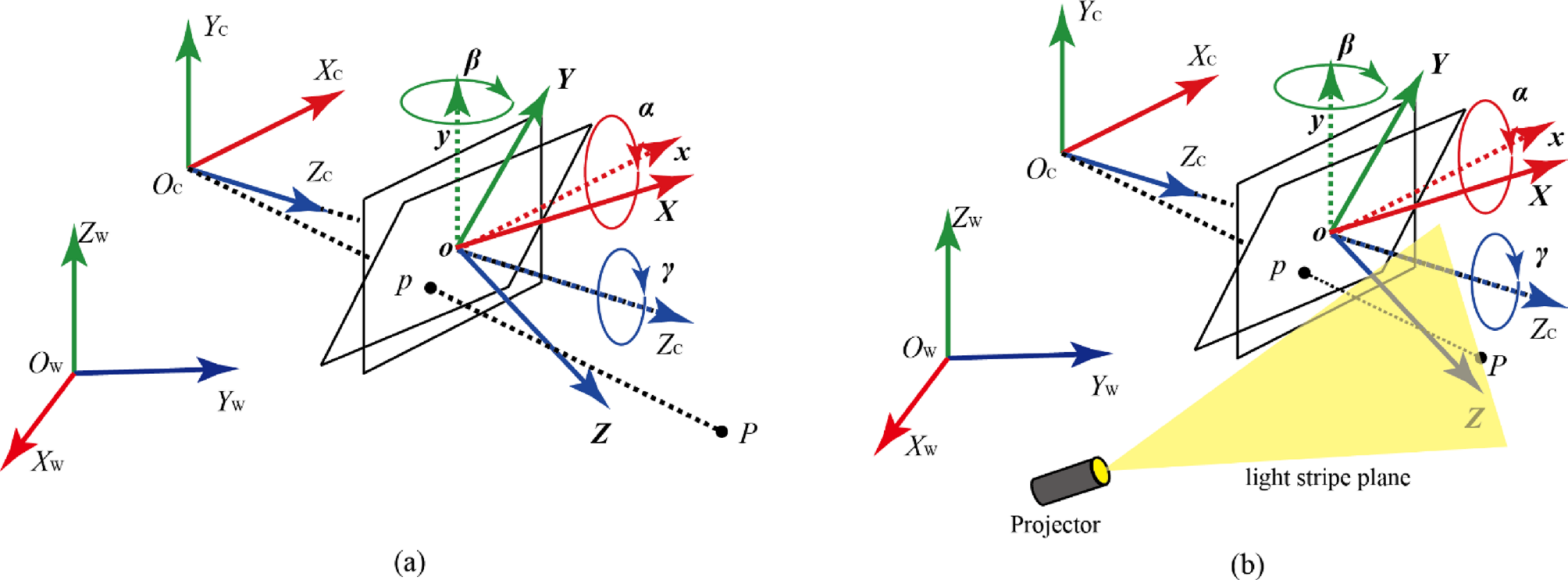
(**a**) Imaging model of a *Scheimpflug* camera, (**b**) Measurement model of a *LSLVS* in *Scheimpflug* conditions.

Imaging model of a *Scheimpflug* camera can be illustrated by Fig. 2. The *O*_C_-*X*_C_*Y*_C_*Z*_C_ is *CCS* while the *o*-*xy* is the *ICS* which is parallel with the lens plane. Meanwhile, we can obtain a 3D coordinate system $o{ - }xyZ_{c}$. When the lens is tilted, $o{ - }xyZ_{c}$ is transformed to *o*-*XYZ*. The transformation matrix can be defined as *R*. Under *CCS*, the camera center is at the origin and the original optical axis points in the positive *Z*_C_ direction. When the lens is tilted, the optical axis is transformed to *Z* direction. A spatial point P is projected to the plane *o*-*XY*, referred to as the real image plane under CCS. *f*_0_ ($\left| {oO_{C} \, } \right|$) is the effective focal length (*EFL*). Supposing $p = \left( {x,y,1} \right)^{{\text{T}}}$ is the projection of $P = \left( {X,Y,Z} \right)^{{\text{T}}}$ on the image plane. Under the idealized pinhole imaging model, i.e. the ideal model of the camera, *P*, *p* and the camera center *O* are collinear^14,16^.

For the related *LSLVS*, structure satisfies the *Scheimpflug* condition, i.e., the image plane, the lens plane and light plane intersect in the *Scheimpflug* line theoretically. As is known, expression of the lens plane under the *CCS* is the *Z*-plane. The light plane can be expressed as

1 $$s_{\beta } c_{\alpha } X - s_{\beta }Y + 2c_{\beta } c_{\alpha } Z - c_{\beta } c_{\alpha } f = 0$$

where $c_{\theta } = \cos \theta$ and $s_{\theta } = \sin \theta$. *α* is the rotation angle around *x*-axis, while *β* is the rotation angle around *y*-axis and *γ* is the rotation angle around *z*-axis.

## Related method

### Feature points on planar target

**Figure 3:**
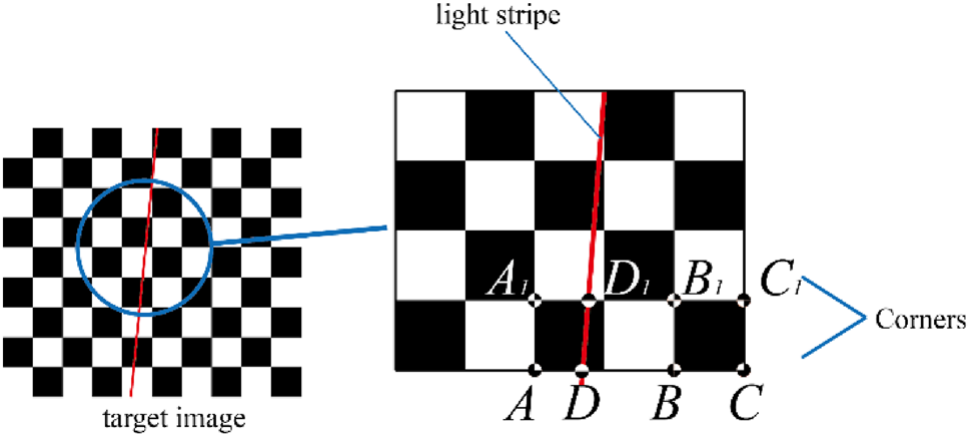
Determination of feature points on planar target.

As is known, the most commonly used planar target is with a checkerboard pattern, which is used to calibrate the camera intrinsic parameters using *Zhang*’s method^17^. When a laser stripe projected on the target, we can get the intersection of light stripe and side of each checker on the image plane (as point *D* and point *D*_1_ in Fig. 3).

As the side length of each checkerboard is known exactly, the coordinate of the feature points under target coordinate system (*TCS*) can be solved based on the invariance of cross-ratio. The theory is described as follow:

The grid pitch of the target is known accurately as *l* while the length of *AD* can be defined as *l*_0_ (see Fig. 3). Based on the invariance of cross-ratio, the following equation can be obtained:

2 $$\frac{{{{AB} \mathord{\left/ {\vphantom {{AB} {BD}}} \right. \kern-0pt} {BD}}}}{{{{AC} \mathord{\left/ {\vphantom {{AC} {DC}}} \right. \kern-0pt} {DC}}}} = \frac{{{{2l} \mathord{\left/ {\vphantom {{2l} {(2l - l_{0} )}}} \right. \kern-0pt} {(2l - l_{0} )}}}}{{{{3l} \mathord{\left/ {\vphantom {{3l} {(3l - l_{0} )}}} \right. \kern-0pt} {(3l - l_{0} )}}}}$$

The real length of *AD* can be solved, so can *A*_1_*D*_1_. Then the distance between point *D* and point *D*_1_ can be worked out. In this case, coordinates of point *D* and point *D*_1_ under *TCS* can be confirmed. Moreover, any one of feature points between point *D* and point *D*_1_ can be calculated out according to related interpolation algorithm.

### Steger’s extraction algorithm

Steger’s algorithm is an extraction algorithm with a sub-pixel precision. It can be used to extract the centerline of a curvilinear structure, such as a laser stripe.

Define the 2D gray-value distribution function of the image as *I*(*X*, *Y*), where (*X*, *Y*) are the coordinates of the image point. The variable quantity of gray values in the position of (*X*, *Y*) is defined as Δ while the change of direction is defined as *n*. *n* and Δ can be determined by Hessian Matrix of *I*(*X*, *Y*). The Hessian Matrix is defined as^18^

3 $$Hess(I(X,Y)) = \left[ {\begin{array}{*{20}l} {I_{XX} } & {I_{XY} } \\ {I_{XY} } & {I_{YY} } \\ \end{array} } \right]$$

where *Hess* indicates numeration of a Hessian Matrix, and the other related notations are defined as below:

4 $$\left\{ {\begin{array}{*{20}c} {I_{XX} = I(X,Y) \otimes g_{xx} (x,y)} \\ {I_{XY} = I(X,Y) \otimes g_{xy} (x,y)} \\ {I_{YY} = I(X,Y) \otimes g_{yy} (x,y)} \\ \end{array} } \right.,$$

In Eq. (4), *g*_xx_, *g*_xy_ and *g*_yy_ are the second-order differential discrete Gaussian convolution kernel and $\otimes$ denotes the calculation of convolution. These two eigenvalues of the Hessian Matrix denote the maximum and the minimum of the second derivative of I(*X*,*Y*). In other words, the eigenvalues of the Hessian Matrix indicate variation in directions of the acutest change and the smoothest change and their corresponding eigenvectors are the related directions.

The centerline of the curvilinear structure is the position where its first-order derivative is zero. Then the center of the curvilinear structure can be determined with sub-pixel accuracy by the second-order Taylor expansion gray-value distribution function. The center with sub-pixel accuracy can be expressed as:

5 $$(p_{x} ,p_{y} ) = (tn_{x} + x_{0} ,tn_{y} + y_{0} ),$$

where

6 $$t = - \frac{{n_{x} g_{x} + n_{y} g_{y} }}{{n_{x}^{2} g_{xx} + 2n_{x} n_{y} g_{xy} + n_{y}^{2} g_{yy} }},$$

(*x*_0_, *y*_0_) is the center with pixel accuracy, whose direction is determined by the eigenvector of the Hessian Matrix, *g*_*x*_ and *g*_*y*_ are the first-order partial derivative of the gray-value distribution function in position (*x*_0_, *y*_0_), while *g*_*xx*_, *g*_*xy*_, *g*_*yy*_ are the second-order partial derivatives of the gray-value distribution function in position (*x*_0_, *y*_0_)^19^

### Normalized feature points in one direction

When we extract center points of line structure feature (such as light stripe and so on) on the image plane with a sub-pixel precision, coordinates of feature points are not a whole number. In some case, *x*-coordinate/*y*-coordinate of feature point should be normalized to a whole number. This process is named as normalization of feature points in *x*-direction/*y*-direction in this paper.

Define point *I* (*x*, *y*) is the extracted feature point of line structure, point *A* (*x*_A_, *y*_A_)is the closest point to *I* on the left, while point *B* (*x*_B_, *y*_B_) is the closest point to *I* on the right. When we normalized point *I* in *x*-direction, we can get coordinates as

7 $$(x_{norm} ,y_{norm} ) = (x_{round} ,\frac{{(y_{B} - y_{A} )x_{round} + y_{A} x_{B} - y_{B} x_{A} }}{{x_{B} - x_{A} }})$$

Moreover, when we normalized point *I* in *y*-direction, we can get coordinates as

8 $$(x_{norm} ,y_{norm} ) = (\frac{{y_{round} (x_{B} - x_{A} ) - y_{A} x_{B} + y_{B} x_{A} }}{{y_{B} - y_{A} }},y_{round} )$$

### Relation between feature point and distance

When obtained enough feature points in different distances, relation between feature point and the distance can be worked out. Once enough sample points are obtained, we can get the relation according to several algorithms, such as curve fitting algorithm, Back-Propagation (*BP*) neural network algorithm and so on.

① Adaptive curve fitting algorithm

In this section, we use a linear function and a second order function to approximate the relationship. Procedure is detailed as follow:

*Step. 1*. Define a linear function as

9 $$f(x) = ax + b$$

and a second order function as

10 $$g(x) = ax^{2} + bx + c$$

*Step. 2*. Normalized feature points and selected feature points with the same *x*-coordinate/*y*-coordinate as the *x* value. Its corresponding distance is chosen as the *y* value.

*Step. 3*. Using least square fitting method^20^ to calculate coefficients *a* and *b* in Eq. (9). If value of the objective function ε (Eq. 11) is smaller than a threshold (such as 1e−4), coefficients are saved and the linear function is selected as the function to express the relation. Else, go to Step.4.

11 $$\varepsilon { = }\sum\limits_{i = 0}^{N} {\left| {f(x_{i} ) - y_{i} } \right|^{2} }$$

In Eq. (11), *f*(*x*) is defined as Eq. (9), *x*i is the normalized *x*-coordinate/*y*-coordinate, while *y*i is corresponding real distance.

*Step. 4*. Using least square fitting method to calculate coefficients *a*, *b* and *c* in Eq. (10). If value of the objective function κ (Eq. 12) is smaller than ε, the second order function is selected as the function to express the relation.

12 $$\kappa { = }\sum\limits_{i = 0}^{N} {\left| {g(x_{i} ) - y_{i} } \right|^{2} }$$

In Eq. (12), *g*(*x*) is defined as Eq. (10), *x*i and *y*i are defined the same as in Eq. (11).

② BP neural network algorithm^21^

In this section, a *BP* neural network with three layers is chosen to obtain the relation between feature point and distance.

*Step. 1*. Normalized feature points and selected feature points with the same *x*-coordinate/*y*-coordinate as the *x* value. Its corresponding distance is chosen as the *y* value.

*Step. 2*. Initialize BP neural network. Select connection weights and thresholds randomly.

*Step. 3*. According to related algorithm for input and output, re-calculate the output values of hidden layer and output layer. Then update related weights and thresholds.

*Step. 4*. Repeat until the error is less than threshold. Then we can get the relation between feature point and distance.

## Calibration procedure

In traditional calibration method, expression of the laser plane under *CCS* should be obtained. But when these captured images are not sharp enough, the calibration results will involve more error. In this section, a new calibration is proposed, including getting the relationship between image point and horizontal distance and the relationship between image point and perpendicular distance. This calibration method is suitable for both *LSLVS*s in traditional structure and in *Scheimpflug* conditions.

### Preparation

*Step. 1*. Select a suitable linear translation stage. As is known, positional precision of a linear translation stage is high enough to calibrate any one laser scanner. In this case, we can choose a suitable linear stage according to the accuracy requirement of our *LSLVS*.

*Step. 2*. Machine a surface plate with a proper precision. As the plate is not with any pattern or 3D feature, productive process is not difficult.

*Step. 3*. A *LSLVS* in traditional structure includes a camera with normal lens and a laser projector. The sensor is fixed on a rigid beam. In this case, the relationship between the camera and the laser projector is unchangeable.

### For perpendicular distance

*Step. 1*. Fix our *LSLVS* on the stage. In this case, the LSLVS can move up and down along the moving direction of the linear translation stage. The translation value is same with the stage, which is easy to obtain.

*Step. 2*. Place the surface plate under the laser projector to cover the measurement range and fix. In this case, image of the laser stripe on the plate can be captured by the camera.

*Step. 3*. Control the linear translation stage moving within the longitudinal measurement range with a fixed step, which is normally equal to the measurement resolution. Capture one image of light stripe for each position.

*Step. 4*. Extract center points of light stripe in each image. In this paper, the extraction method is *Steger*’s extraction algorithm^19^ with a precision of subpixel.

*Step. 5*. Normalized image feature points. Then get the function between coordinate of center points and perpendicular distance in each position according to related method.

### For horizontal distance

*Step. 1*. Fix our *LSLVS* on the stage. In this case, the LSLVS can move up and down along the moving direction of the linear translation stage.

*Step. 2*. Place a planar target with checkerboard-pattern on the surface plate.

*Step. 3*. Control the linear translation stage moving within the longitudinal measurement range with a fixed step. Then capture one image of light stripe (with the planar target) for each position.

*Step. 4*. Extract center points of light stripe in each image. In this paper, the extraction method is *Steger*’s extraction algorithm^19^ with a precision of subpixel.

*Step. 5*. Normalized image feature points according to normalization algorithm mentioned in Part *B* of Section III. Then get the function between coordinate of center points and horizontal distance in each position according to related method.

## System structure

**Figure 4:**
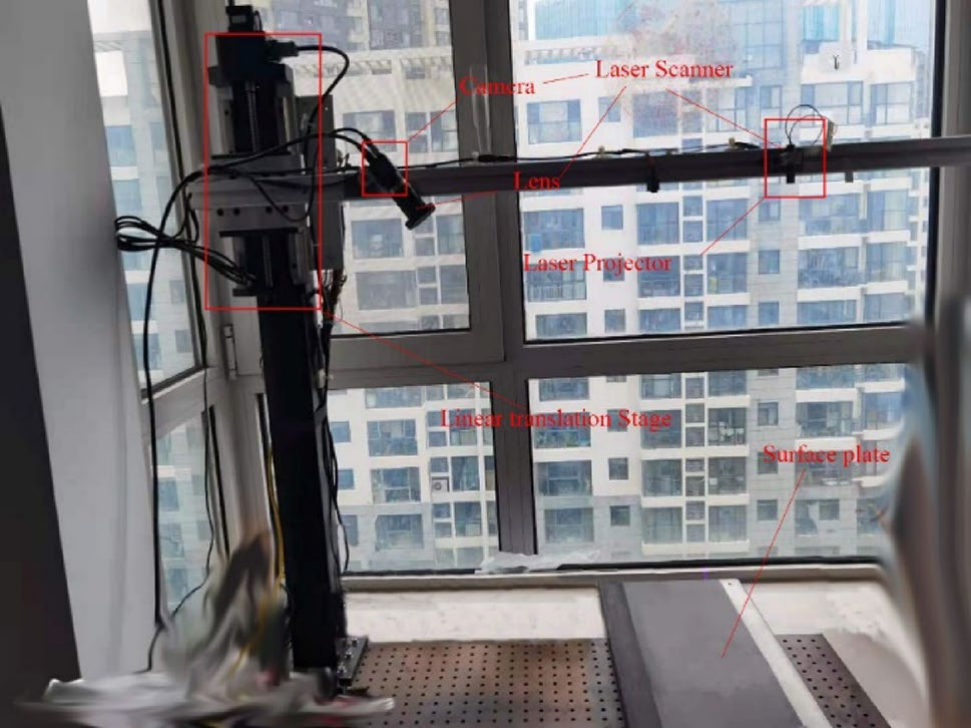
Construct of our experiment apparatus.

The structure of our experiment apparatus is illustrated in Fig. 4. For simplicity, we built a *LSLVS* with a traditional structure in our work, which includes a camera with a normal lens and a laser projector (Note that a LSLVS in *Scheimpflug* condition can also be utilized). The sensor is fixed on a rigid beam. In this case, the relationship between the camera and the laser projector is unchangeable. Then the beam is fixed on a linear translation stage. Components are detailed as following.

### LSLVS

The camera used in our *LSLVS* is *DaHeng MER-1070-10GM-P* with a resolution of 3840 × 2748 pixels while the laser projector is with a wavelength of 405 nm. In practice, the resolution in *x*-direction is reduce to 3300 pixels to make sure a whole light stripe is captured. Baseline between the camera and the laser projector is about 500 mm. As the focal length of our lens is 6 mm, measurement range is about 1100 mm while the nearest perpendicular distance from the rigid beam to the reference plane is about 800 mm.

### Linear translation stage

The stage used to calibrate the *LSLVS* is chosen as PST150 X-S42 with a stroke of 150 mm. Resolution of the stage is 2.5 us, while the repositioning precision is 4 us. Positional accuracy of the linear translation stage is with a high enough precision according to the measurement requirement of the *LSLVS*. As the measurement range is 1100 mm, we selected the aimed precision as 0.1 mm, i.e., 0.09‰ relative to the measurement range, which is better than most existing sensors in *Scheimpflug* conditions.

### Surface plate

Precision of the planeness is 0.05 mm and the length is 1200 mm. As the plate is not with any pattern or 3D feature, precise machining is easy to finish. In the process of calibration, the *LSLVS* projects a laser stripe onto the surface plate, and the camera captures images of the laser stripe.

### Planar target

**Figure 5:**
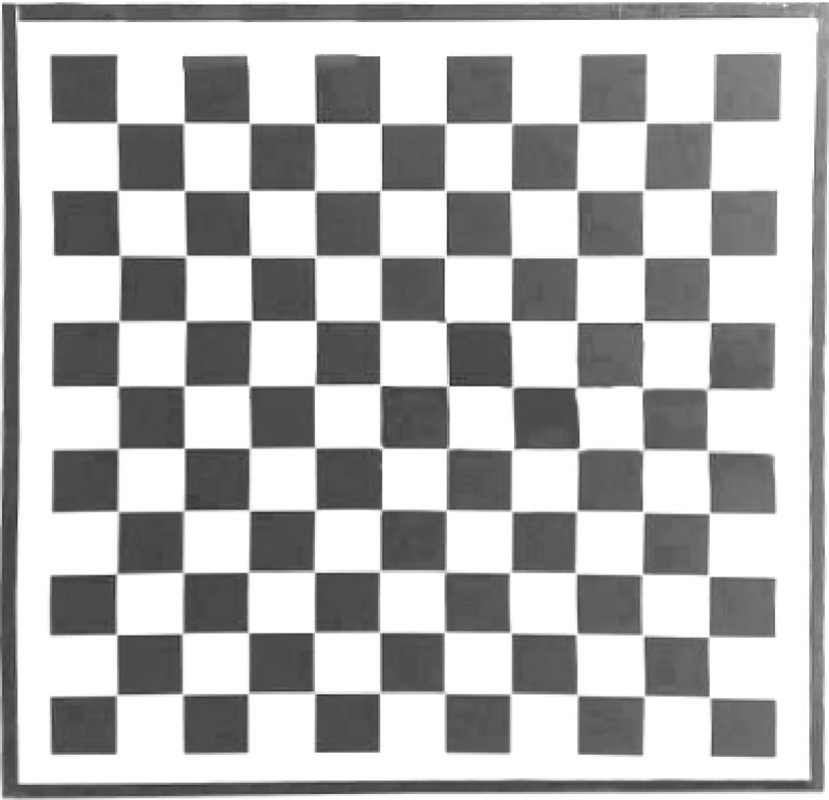
The planar target with checkerboard pattern.

The planar target used to get feature points on light stripe is with a checkerboard pattern (as illustrated in Fig. 5). Machining precision of the planar target is 5 μm. The side length of each checkerboard is 25 mm. The surface of the planar target is diffusely reflected. In this case, feature points on light plane can be obtained easily.

## Experiments and discussion

### Camera calibration

**Figure 6:**
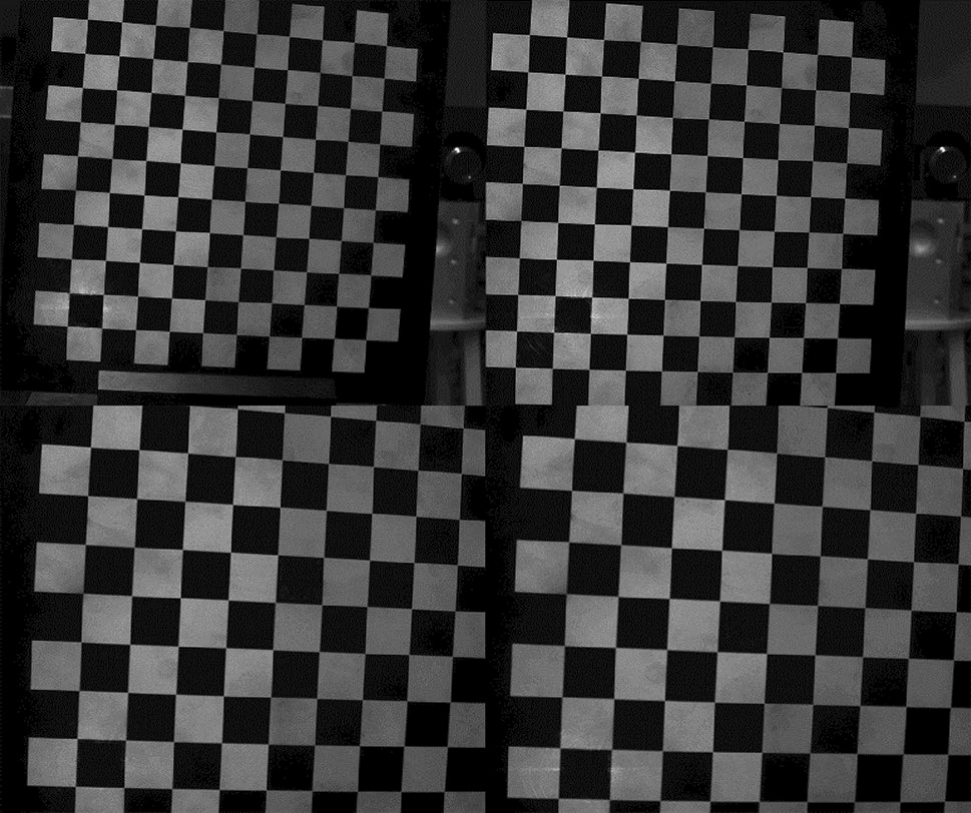
Part of target images used to calibrate the camera.

With the help of the planar target mentioned above, camera used in the system is calibrated by *Zhang*’s calibration method^17^. In this method, the target is located in camera’s field of view randomly. In this case, images of the target can be captured by the camera sharply. When the target is located into more than three different positions with different poses, the camera can be calibrated successfully. In our paper, sixteen images are captured to finish the calibration task and part of images are illustrated in Fig. 6.

Obtained intrinsic parameters are listed in Table 1.

**Table 1:** Intrinsic parameters of the camera in our system.

*f*_x_	*f*_y_	*u*_0_	*v*_0_	*kc*_1_	*kc*_2_
3744.76	3754.28	1625.68	223.19	− 0.130	0.026

In Table 1, $f_{x}$ and $f_{y}$ are scale factors in *x*-coordinate and *y*-coordinate, and $(u_{0} ,v_{0} )$ are the principal point of the image plane. *kc*_1_ and *kc*_2_ are distortion factors of the image.

### Calibration of LSLVS

In the calibration for our *LSLVS*, the fixed step of the linear translation stage is 0.1 mm while the longitudinal measurement range is 100 mm. In this case, we should capture 1001 images to get the relation between feature points and perpendicular distance. Then the planar target is located on the surface plane to get the relation between feature points and horizontal distance. For each image, the light stripe is extracted by *Steger*’s method^19^ and then feature points are normalized by method mentioned in Section II.

**Figure 7:**
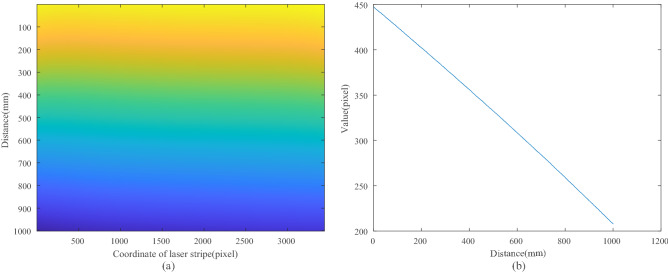
(**a**) relationship between distance and coordinate of laser stripe; (**b**) relationship between the value and the distance in one position of the laser stripe.

As the resolution of the image on *x*-direction is 3300, we should get 3300 functions to express relationship for each pixel in whole measurement range. Relationship between distance and coordinate of laser stripe are illustrated in Fig. 7a. As illustrated in Fig. 7a, each column is variation of distance with the same *x*-coordinate of feature points. In other words, each column denotes function of distance with *y*-coordinate. Sample of relationship between the *y*-coordinate and the distance in one position of the laser stripe is illustrated in Fig. 7b. From Fig. 7b, the relationship can be confirmed by two algorithms, i.e., the adaptive curve fitting algorithm and BP neural network algorithm. In the adaptive curve fitting algorithm, the each relationship (as plotted in Fig. 7b) is fitted by a linear function as Eq. (9) or a second order function as Eq. (10), while in the BP neural network algorithm the relationship of input and output is expressed by a trained network model.

### Evaluations

① For perpendicular distance

**Figure 8:**
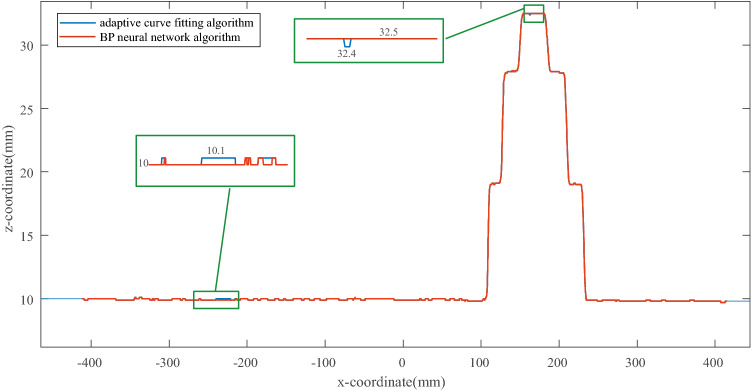
Measurement results to evaluate the measurement precision of our LSLVS.

In order to evaluate the measurement precision of the *LSLVS* in perpendicular direction according to our proposed approach, we produce a step wedge with a precision of 0.02 mm. Heights of these step surfaces are 10.0 mm, 19.0 mm, 27.9 mm and 32.5 mm respectively. The wedge is located on a surface plate. These measurement results are illustrated as Fig. 8. From these measurement results we can get maximum of the error as 0.1 mm. As the fixed step of the linear translation stage used to get the relationship is 0.1 mm, measurement precision of our laser scanner is precise enough.

② For horizontal distance

**Figure 9:**
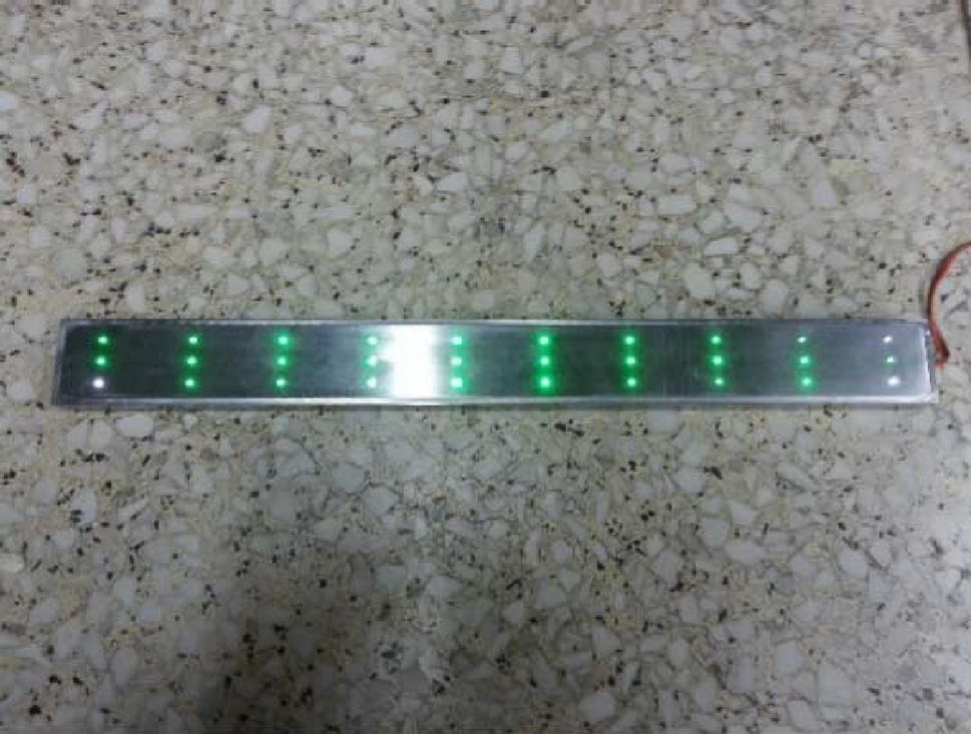
1D target to evaluate our measurement method in horizontal direction.

A 1D target which is illustrated in Fig. 9, is used to evaluate the measurement precision of the laser scanner in horizontal direction. The distance between each two adjacent feature points is known exactly (40 mm). All measurement values are compared with their corresponding true value. As the target can be moved into different positions randomly, we can obtain enough distances to evaluate our measurement method in horizontal direction. Part of our evaluation results are listed in Table 2. In Table 2, *D*_m1_ denotes the measurement result according to adaptive curve fitting algorithm, while *D*_m2_ denotes measurement result according to BP neural network algorithm. Correspondingly, Error1 is the absolute error of *D*_m1_ and Error2 is the absolute error of *D*_m2_. As listed in Table 2, the root mean square error (*RMS* error) of measurement results according to our proposed method is 0.065 mm (according to adaptive curve fitting algorithm) and 0.052 mm (according to BP neural network algorithm).

**Table 2:** Result of the evaluation (unit:mm).

No	*D*_real_	*D*_m1_	*D*_m2_	Error1	Error2
1	40.00	40.08	40.06	0.08	0.06
2	40.00	39.92	39.93	− 0.08	− 0.07
3	40.00	39.90	39.92	− 0.10	− 0.08
4	40.00	39.97	39.95	− 0.03	− 0.05
5	40.00	40.05	40.06	0.05	0.06
6	40.00	39.96	39.97	− 0.04	− 0.03
7	40.00	40.03	40.04	0.03	0.04
8	40.00	39.96	39.98	− 0.04	− 0.02
9	40.00	39.91	39.96	− 0.09	− 0.04
10	40.00	40.06	40.04	0.06	0.04
RMS				0.065	0.052

### Comparison

For comparison, we calibrate the *LSLVS* using Wei’s calibration method^9,10^. The expression under *CCS* of the light plane is

13 $$- 0.014X - 0.793Y + 0.608Z - 581.157 = 0$$

we measure the 1D target illustrated in Fig. 8 according to the traditional method ($D_{trad}$) and our proposed method (*D*m1 and *D*m2). The measurement results are listed in Table 3.

**Table 3:** Comparison results of measurement.

No	*D*_real_(mm)	*D*_m1_	*D*_m2_	*D*_trad_(mm)
1	40.00	40.08	40.06	40.15
2	40.00	39.92	39.93	40.11
3	40.00	39.90	39.92	39.82
4	40.00	39.97	39.95	39.93
5	40.00	40.05	40.06	40.20
6	40.00	39.96	39.97	39.78
7	40.00	40.03	40.04	39.83
8	40.00	39.96	39.98	39.75
9	40.00	39.91	39.96	40.24
10	40.00	40.06	40.04	40.17
RMS		0.065	0.052	0.184

As listed in Table 3, *RMS* error of measurement results according to the traditional method is 0.184 mm, which is at least reduced by 64.67%.

## Conclusion

In this paper, we propose a precise measurement method for a line structure light vision sensor, including corresponding calibration method. Related procedure is detailed in this paper. Compared to a traditional measurement method, distortion compensation is not necessary and precision of measurement is improved. Moreover, we demonstrated the error of our present measurement method in a large measurement range (about 1100 mm) is 0.1 mm, i.e., 0.09‰ relative to the measurement range. Real experiments show that root mean square error of measurement results according to our proposed method is reduced by 64.67% related to the traditional method. Moreover, our proposed method can also be used for the line structure light vision sensor in *Scheimpflug* conditions.

## Data Availability

All data generated or analysed during this study are included in this published article.
